# Mycophenolate Mofetil (CellCept®) in Combination With Low Dose Prednisolone in Moderate to Severe Graves' Orbitopathy

**DOI:** 10.3389/fmed.2022.788228

**Published:** 2022-02-11

**Authors:** Mohammad Taher Rajabi, Seyed Mohsen Rafizadeh, Abbas Mohammadi, Bahram Eshraghi, Nader Mohammadi, Seyedeh Simindokht Hosseini, Mohammad Bagher Rajabi, Mohammad Mohsen Keshmirshekan, Mansoor Shahriari, Seyedeh Zahra Poursayed Lazarjani, Mohammad Mehdi Parandin

**Affiliations:** ^1^Eye Research Center, Farabi Eye Hospital, Tehran University of Medical Sciences, Tehran, Iran; ^2^Department of Eye, Eye Research Center, Amiralmomenin Hospital, School of Medicin, Guilan University of Medical Science, Rasht, Iran

**Keywords:** Graves' orbitopathy, prednisolone, CellCept®, mycophenolate mofetil (MMF), thyroid eye disease (TED)

## Abstract

Although corticosteroids are currently the first-choice drug for thyroid eye disease (TED), in 20–30% of cases, patients show poor or non-existent responses, and when the drug is withdrawn, 10–20% of patients relapse. Thus, in this study, we aimed to investigate the efficacy of the combined use of mycophenolate mofetil (CellCept®) and low dose oral prednisolone in patients with moderate to severe Graves' orbitopathy (GO). For the first time, we investigated the relationship between TED-related parameters and proptosis reduction. In a prospective, non-randomized, interventional case series, 242 patients with moderate-to-severe GO were, assigned to receive oral prednisolone (5 mg/ d) and mycophenolate mofetil (CellCept®) (one 500 mg tablet twice per day according to the therapeutic response). The patients were monitored regularly during the 3rd, 6th, 12th, and 18th month of treatment. The main outcome measures were the clinical activity score (CAS), intraocular pressure (IOP), diplopia, proptosis and visual acuity. We also assessed the relationship between the main outcomes with proptosis changes and time to improvement (months). Adverse effects were recorded during each visit. The clinical response rate increased from 67.7% on the third month to 89.2% on the sixth month, and 94.2% on the 12th month. This therapeutic response continued until the 18th month of follow-up. The CAS responses [disease inactivation (CAS <3)] improved during our study: 70.6% on the third month, 90.0% on the sixth month, and 92.5% at 12th month. These conditions continued until the 18th month of follow-up. Proptosis improvement was 52% on the third month, 71% on the sixth month, 83% on the 12th month, and 87.1% on the 18th month. Changes in IOP and visual acuity were not significant (*P* = 0.568 and 0.668, respectively). The patient showed significant improvement in the Gorman score. A Shorter duration of treatment was seen in patients with earlier onset of intervention, younger age, and lack of all extraocular muscle (EOM) enlargement on computed tomography (CT) scan (*p* < 0.05). In addition, a better response (more reduction) in proptosis was related to: younger age at disease, earlier treatment intervention (less interval from the time the diagnosis of moderate-to-severe GO was made until medication initiation), shorter treatment time (less time to improvement), less IOP, lack of EOM enlargement on CT scan, and lack of diplopia (*P* < 0.05). Adverse events occurred in six patients. Findings show that mycophenolate mofetil (CellCept®) plus low-dose prednisolone can be introduced as a new optimal dosing regimen in GO due to its better effect on chronic complications such as proptosis and diplopia.

## Introduction

Graves' orbitopathy (GO) is an inflammatory disorder of the orbit that occurs more frequently in females than in males and in the middle age than in other age groups. Moreover, GO is associated with autoimmune thyroid disease (1). Although the etiology of GO is unknown, the role of B and T lymphocytes has been established, and histological examination has shown infiltration of activated B cells, T cells, and macrophages, and expansion of orbital fat tissues and extraocular muscle (EOM) (2). Unfortunately, the medical treatment of GO remains a big problem and challenge in some patients.

In a much as 20–60% of patients with active GO, medical treatment with oral and/or intravenous steroids may fail and mainly depend on the form of administration and dosage, and these patients suffer recurrence and experience significant adverse effects (3–5). Due to these limitations, new treatments that directly target pathogenic mechanisms of GO have long been sought (6).

Numerous new treatments using novel immunomodulatory drugs, such as rituximab have been introduced to modulate inflammatory disorders but in (GO), immunosuppressive drugs such as methotrexate, tumor necrosis factor (TNF)-alpha inhibitors, or azathioprine have limited effects on the long-term sequelae of thyroid eye disease, such as disability from proptosis and diplopia, which impair quality of life (7–9).

Mycophenolate mofetil (MMF) is a selective, reversible and competitive inhibitor of inosine monophosphate dehydrogenase (IMPDH) that is used in combination with cyclosporine and corticosteroids to prevent graft rejection subsequent after stem cell or solid organ transplantation. Metabolite mycophenolic acid (MPA) is the prodrug of MMF that is hydrolyzed by esterases. Metabolite mycophenolic acid inhibits inosine monophosphate dehydrogenase (IMPD), leading to the inhibition of the *de novo* pathway for guanosine monophosphate (GMP) synthesis, which suppresses the *de novo* synthesis of purines, primarily blocking proliferation and inducing the apoptosis of activated B and T lymphocytes. In addition, MPA by guanosine-tri-phosphate (GTP) depletion leads to decreased expression of adhesion molecules and modulation of the chemotaxis of activated lymphocytes in inflammatory tissues (10, 11).

Due to immune modulatory effect, MMF has been evaluated and used in various autoimmune diseases; however, the results of using MMF in GO have been very variable and performed in patient with moderate to severe GO and on a low sample size. Thus, in this study, we aimed to assess the efficacy and tolerability of combined therapy with MMF (CellCept®) and low doses oral prednisolone in GO and provide the basis for further studies to introduce a new regimen for moderate to severe thyroid eye disease (TED).

## Patients and Methods

In a prospective, non-randomized, interventional case series, 261 patients who were diagnosed with GO, between September 29, 2014 and July 31, 2018 in the Farabi Eye Hospital were enrolled in this study. Approval for the study was obtained from the ethical committee of the Tehran University of Medical Sciences which is in compliance with the Helsinki Declaration. All patients received a thorough explanation of the study design and aims, and were provided with written informed consent.

The patients enrolled in this study were: (1) 18–60 years old; (2) diagnosed with hyperthyroidism and were on antithyroid therapy or diagnosed with euthyroidism who previously used antithyroid therapy; and (3) diagnosed with active TED [clinical activity score ≥ 3: gaze-provoked orbital pain, orbital pain at rest, lid swelling, lid redness, conjunctiva redness, chemosis, or swelling of the caruncle (12)] that was moderate to severe [according to the European Group on Graves' Orbitopathy (EUGOGO) color atlas evaluation (13)]. The patients should not have previously received corticosteroid or immunosuppressive treatment for GO or any reason within the past 3 months. Patients with impaired heart, kidney, and liver function, intraocular pressure (IOP) above 40 mm/Hg, other inflammatory diseases of the orbit, sinusitis, optic neuropathy, exposure keratopathy (EK), orbital radiotherapy and any relevant malignancy were excluded from this study.

Oral prednisolone combined with mycophenolate mofetil (CellCept®) was administrated to all patients. Oral prednisolone was started at dose of 20 mg and was gradually tapered, with a reduction of 5 mg per week for 3 weeks and then continued at a dose of 5 mg (orally once a day). Patients also received 500 mg of mycophenolate mofetil (CellCept®) orally twice per day which is a commonly used dose in patients with autoimmune diseases and continued depending on the clinical response. This regimen was continued for 3 months after clinical response, and then discontinued in such a way that, prednisolone was abruptly discontinued and CellCept® was gradually tapered, with a reduction of 500 mg for 1 month (orally one per day), and then discontinued.

All patients underwent complete ophthalmic at baseline and at 3, 6, 12, and 18 months after starting the treatment, and monthly endocrine assessments. Ophthalmic assessment was performed by a single ophthalmologist, using a researcher-made form, including soft tissue involvement (inflammatory eyelid swelling), clinical activity score (seven items) (12), proptosis in mm (measured using a Hertel exophthalmometer), diplopia [assessed using the Gorman diplopia score: no diplopia (absent), diplopia when the patient was tired or awakening (intermittent), diplopia at extremes of gaze (inconstant) and continuous diplopia in the primary and reading position (constant)], visual acuity using the Snellen chart in decimals, and IOP using the Goldmann applanation tonometer. At the beginning of the study, all patients underwent spiral orbital computed tomography (CT) scans to assess the size and enlargement of extraocular muscles.

The primary outcome was the overall response at the 3rd, 6th, 12th, and 18th month. Response was defined as the reduction of at least two measures in the composite index in at least one eye, without deterioration in any of the same measures in either eye; that is, improvement in soft tissue involvement by one grade in any of the following: eyelid swelling, eyelid erythema, conjunctival redness, or conjunctival edema; improvement in clinical activity score by at least 2 points or more points or disease inactivation, improvement in proptosis of at least 2 mm, improvement in vertical palpebral fissure of at least 2 mm, improvement in diplopia (disappearance or change in the degree), increase in visual acuity ≥ 2/10 and IOP changes.

The tolerability and safety of this regimen were assessed by recording the adverse events, physical examinations and laboratory parameters. At each visit, all patients were questioned about any adverse events. Adverse events were defined as any undesirable symptoms or sign that occurred after the initiation of treatment, regardless of its relation to the study drug. A serious adverse event was defined as any untoward medical occurrence that, at any dose, was life-threatening, required hospitalization, required medical intervention, or resulted in persistent or significant disability, incapacity, or a congenital malformation. Adverse events were usually monitored by the endocrinologist and did not influence the ophthalmological examination. Laboratory tests including glucose, electrolytes, transaminases, serum lipid and uric acid, kidney function, and body weight were assessed every month.

All statistical analyses were performed using SPSS version 22 (SPSS Inc., Chicago, IL, USA). The mean, median, standard deviation, median, frequency and percentage were used to express the data. A linear mixed model was used to investigate changes in the parameters during the study and changes in proptosis. Kaplan–Meier survival graphs were used to determine the intensity of the treatment. *P* < 0.05 indicated a statistically significant difference.

## Results

A total of 261 patients received MMF in combination with prednisolone. Thirteen patients due to lack of documentation and six patients due to adverse events were excluded from this study. A total of 61.2% (148) of our study were female, and the mean age was 39 ± 12 years old. Of the patients, 20.2% were under 30 years old, 55.4% were between 31 and 45 years old, and 24.4% were over 45 years old. The demographic and clinical data of the patients at baseline are summarized in Table 1. The mean start time of medication (from the time diagnosis of moderate to severe GO was made) was 7 ± 4 months. In 122 patients (50.4%), the start time of medication was <6 months from the time of the GO diagnosis, and in 120 patients, more than 6 months had passed since the onset of the GO before treatment was initiated.

**Table 1:** Baseline demographic and clinical data.

**Parameter**		**Value**
Age	Mean ± standard deviation (SD)	39 ± 12
	≤ 30	49 (20.2%)
	31–45	134 (55.4%)
	46+	59 (24.4%)
Sex	F	148 (61.2%)
	M	94 (38.8%)
Start time of medication [from the time the diagnosis of moderate to severe Graves' orbitopathy (GO) was made]	Mean ± SD	7 ± 4
	≤ 6	122 (50.4%)
	7+	120 (49.6%)
Time to improvement (months)	Mean ± SD	10 ± 6
	Median (range)	9 (2 to 26)
Clinical activity score (CAS)	Mean ± SD	5.4 ± 0.2
Intraocular pressure (IOP)	Mean ± SD	20 ± 4
	Median (range)	18 (10 to 34)
Proptosis	Mean ± SD	21.5 ± 1.8
	Median (range)	22 (17 to 26)
Visual acuity (mean, most affected eye)	Mean ± SD	0.93 ± 0.13
All extraocular muscle (EOM) enlargement	No	196 (81.0%)
	Yes	46 (19.0%)
Conjunctival injection	No	83 (34.3%)
	Yes	159 (65.7%)
Caruncular/plica injection	No	120 (49.6%)
	Yes	122 (50.4%)
Chemosis	No	85 (35.1%)
	Yes	157 (64.9%)
Lid swelling	No	135 (55.8%)
	Yes	107 (44.2%)
Pain	No	158 (65.3%)
	Yes	84 (34.7%)
Retraction	No	87 (36.0%)
	Yes	155 (64.0%)
Diplopia (Gorman)		
Absent	96 (39.6%)	
Intermittent	84 (34.7%)	
Inconstant	44 (18.1%)	
Constant	18 (7.4%)	

**Figure 1:**
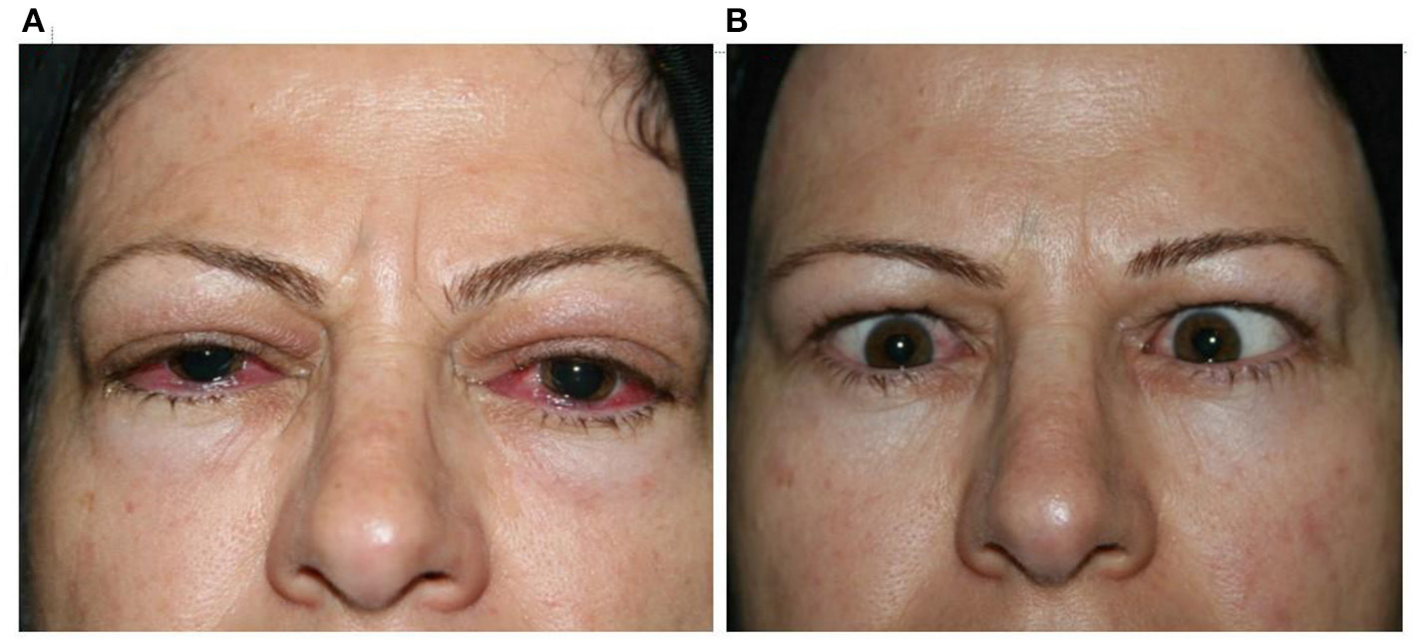
Clinical response 2 months [**(A)** before; **(B)** after] after receiving MMF combined with low-dose prednisolone.

The clinical response rate increased from 67.7% on the third month to 89.2% on the sixth month and 94.2% on the 12th month. This therapeutic response continued until the 18th month of follow-up (Table 2; Figure 1).

**Table 2:** Ophthalmological evaluation during the study.

**Parameter**	**Time**									
	**Baseline**	**Month 3**		**Month 6**		**Month 12**		**Month 18**		***P*-value**
			***P***		***P***		***P***		***P***	
Clinical activity score (CAS; [mean ± standard deviation (SD)]	5.4 ± 0.2	2.8 ± 1.1	<0.001	2.3 ± 1.2	<0.001	1.5 ± 1.2	<0.001	1.2 ± 1.1	<0.001	<0.001
Improved	–	171 (70.6%)		218 (90.0%)		224 (92.5%)		224 (92.5%)		
Unchanged	–	71 (29.4%)		24 (10.0%)		8 (7.5%)		18 (7.5%)		
Worsened	–	0		0		0		0		
Intraocular pressure (IOP, mmHg; mean ± SD)	19.7 ± 4.3	19.5 ± 4.1	0.98	20.0 ± 32.9	0.999	19.5 ± 4.2	0.996	19.6 ± 4.4	>0.99	0.568
**Proptosis (Mean** **±SD)**										
Right eye	21.5 ± 1.8	20.3 ± 1.7	<0.001	19.3± 1.9	<0.001	18.4 ± 2	<0.001	18.06 ± 1.8	<0.001	<0.001
Left eye	21.31 ± 1.25	20.19 ± 1.57	<0.001	18.97 ± 1.7	<0.001	18.28 ± 2	<0.001	17.85 ± 1.8	<0.001	
Improved	–	126 (52%)		172 (71%)		201 (83%)		211 (87.1%)		
Unchanged	–	116 (48%)		71 (29%)		41 (17%)		31 (12.9%)		
Worsened	–	0		0		0		0		
**Diplopia (Gorman)**										
Absent	96 (39.6%)	130 (53.7%)	<0.001	182 (75.2%)	<0.001	208 (85%)	<0.001	214 (88.4%)	<0.001	<0.001
Intermittent	84 (34.7%)	70 (28.9%)	0.012	39 (16.1%)	<0.001	16 (6.6%)	<0.001	11 (4.5%)		
Inconstant	44 (18.1%)	32(13.2%)		17 (7%)		14 (5.7%)		13 (5.3%)		
Constant	18 (7.4%)	10 (4.1%)		4 (1.6%)		4 (1.6%)		4 (1.6%)		
Improved	–	179 (73.9%)		212 (87.6%)		228 (94.21%)		231 (95.4%)		
Unchanged	–	63 (26.03%)		27 (11.15%)		14 (5.78%)		11 (4.6%)		
Worsened	–	0		3 (1.2%)		0				
Visual Acuity (mean ± SD)	0.93 ± 0.13	0.92 ± 0.14	0.999	0.92 ± 0.12	0.985	0.94 ± 0.13	0.898	0.95 ± 0.11	0.777	0.668
Response	–	164 (67.7%)		216 (89.2%)		228 (94.2%)		228 (94.2%)		

The mean clinical activity score (CAS) decreased significantly from a baseline of 5.4 ± 0.2 to 2.8 ± 1.1 on the third month, to 2.3 ± 1.2 on the sixth month, to 1.5 ± 1.2 on the 12th month, and 1.2 ± 1.1 on the 18th month (*P* < 0.001). Furthermore, the CAS responses [disease inactivation (CAS <3)] improved during our study: 70.6% on the third month, 90.0% on the sixth month, and 92.5% on the 12th month. These conditions continued until the 18th month of follow-up (Table 2).

Similarly, the proptosis response improved: by 52% on the third month, by 71% on the sixth month, by 83% on the 12th month, and by 87.1% at 18th month. The mean proptosis values decreased significantly at 3 and 6 months (Table 2).

During the treatment, there was no evidence of an increase in IOP, so that, IOP was 19.7 ± 4.3 at baseline, 19.5 ± 4.1 on the third month, 20.0 ± 32.9 on the six month, 19.5 ± 4.2 on the 12th month and 19.6 ± 4.4 on the 18th month, and these changes were not statistically significant (*P*: 0.568; Table 2).

The visual acuity changes were 0.93 ± 0.13 at baseline, 0.92 ± 0.14 on the third month, 0.92 ± 0.12 on the sixth month, 0.94 ± 0.13 on the 12th month, and 0.95 ± 0.11 on the 18th month, and the differences were not significant (*P*: 0.668; Table 2).

Diplopia was significantly improved in 179 patients (73.9%) at 3 months, 212 patients (87.6%) at 6 months, 228 patients (94.21%) at 12th months, and 231 patients (95.4%) at 18th months (*P* < 0.001). At baseline, absent, intermittent, inconstant and constant diplopia was present in 96 (39.6%), 84 (34.7%), 44 (18.1%) and 18 (7.4%) patients, respectively. Following treatment, diplopia not observed in 214 (88.4%) patients, while 11 (4.5%), 13 (5.3%), and 4 (1.6%) patients had intermittent, inconstant, and constant diplopia, respectively (*P* < 0.01 vs. before treatment; Table 2).

**Figure 2:**
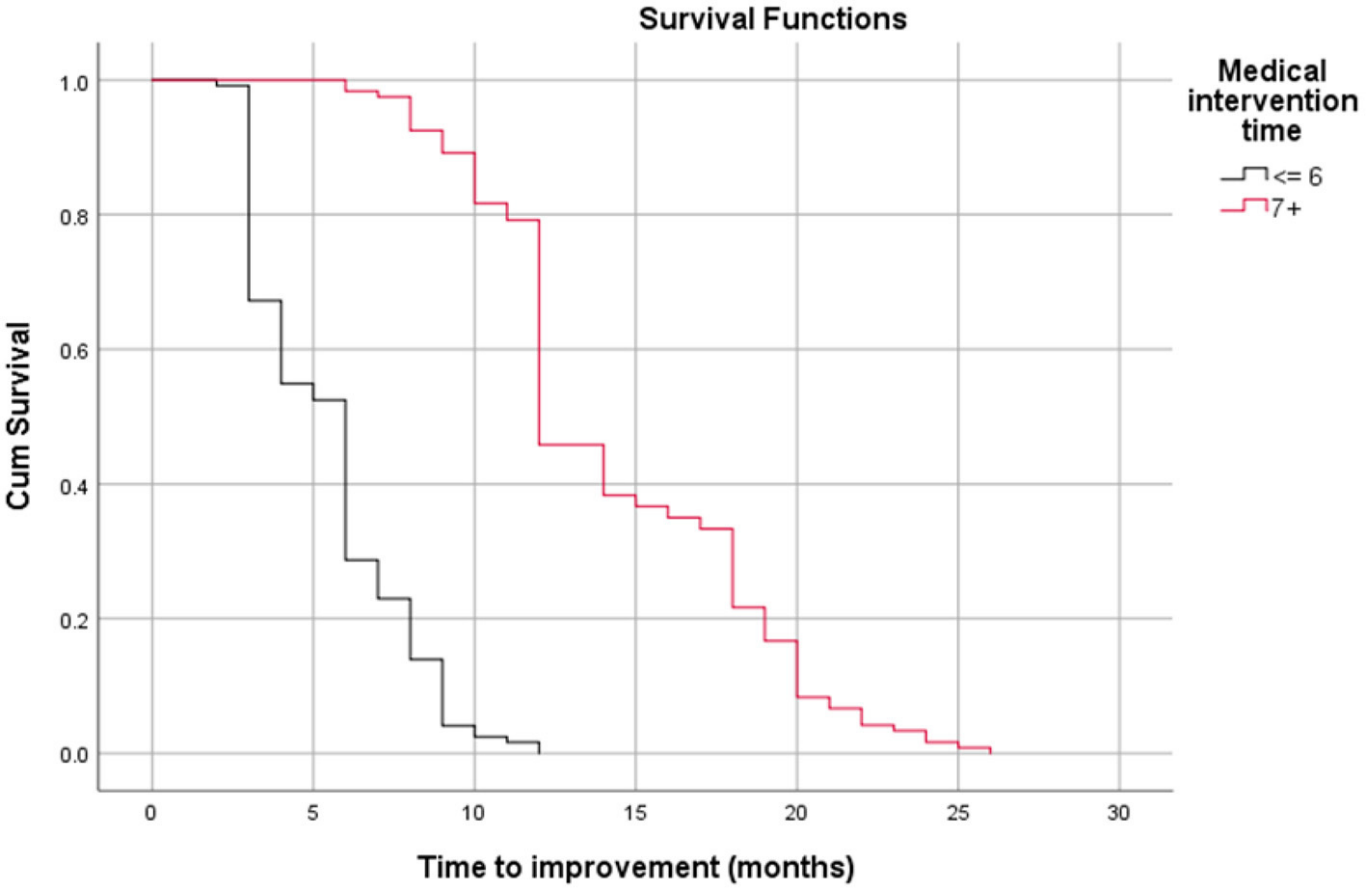
Relationship between time to improvement and medical intervention time (start time) (log-rank test, *p* < 0.05).

**Figure 3:**
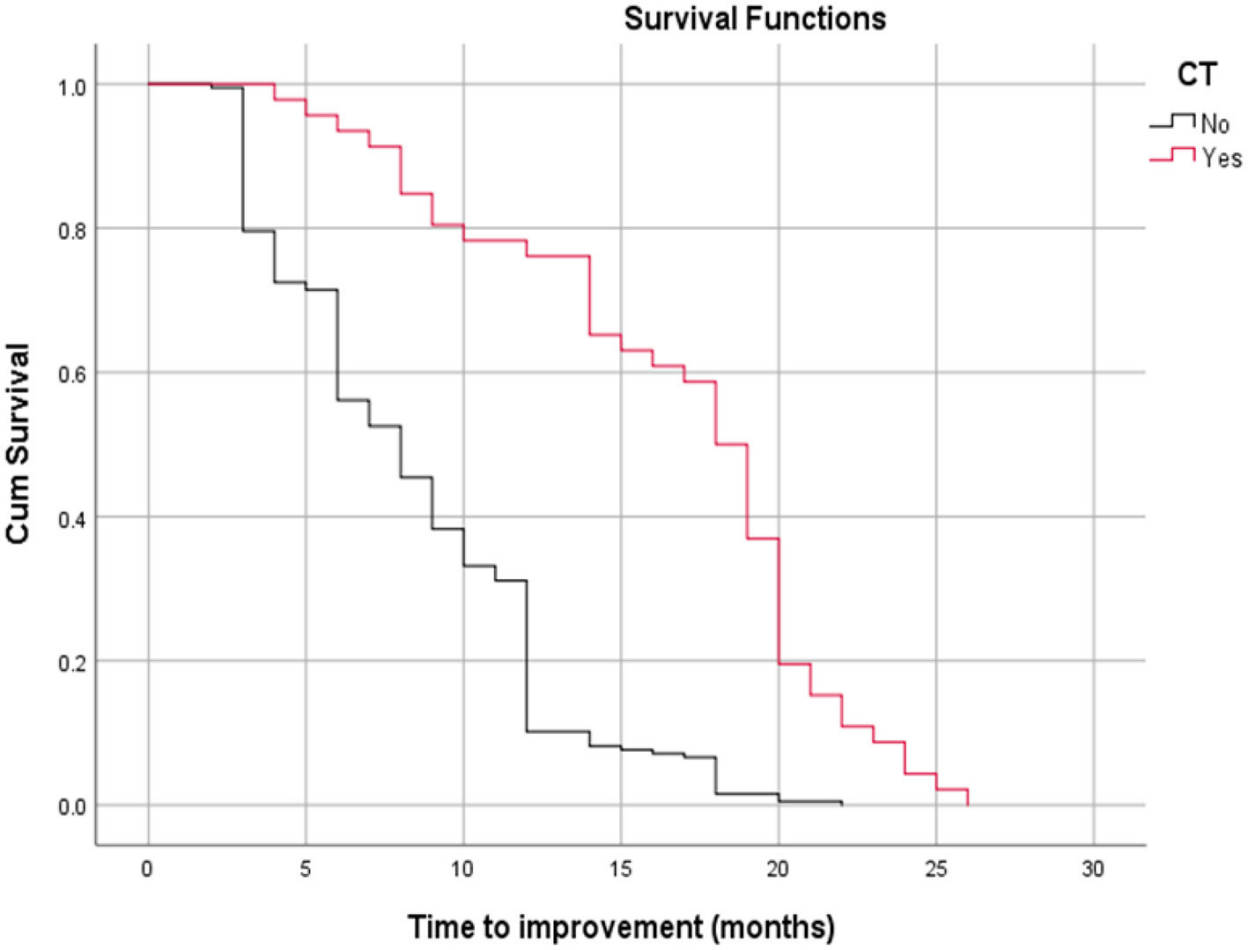
Relationship between time to improvement with all extra ocular muscle's enlargement on a CT scan (log-rank test, *p* < 0.05).

Table 3 shows the relationship between time to improvement and other study parameters. Patients who started medication earlier (<6 months compared to over 6 months); in other words, those who had a medical intervention time <6 months, had a younger age at diagnosis of the disease, and had a lack of all EOM enlargement responded faster to treatment (*P* < 0.05); however, age was not a significant factor in the multivariate analysis (Table 3). Furthermore, the median time to improvement in the case of medical intervention time <6 months was 6 months, while if the medical intervention time was more than 6 months, the time to improvement reached to 12 months (*P* < 0.05; Figure 2). Moreover, if all EOMs on the CT scan were not large, the median time to improvement was 8 months, whereas if all the muscles on the CT scan were large, the time to improvement was 12 months (*P* < 0.05; Figure 3; Table 4).

**Table 3:** Relationship between time to improvement (as the time it takes for patients to recover) with other study parameters.

**Parameter**	**Level**	**Univariate**				**Multivariable**			
		**HR**	**95% confidence interval (CI)**		**P**	**AHR**	**95% CI**		**P**
			**Lower**	**Upper**			**Lower**	**Upper**	
Medical intervention time (from the time the diagnosis was made)	≤ 6	1.00				1.00			
	7+	11.80	8.07	17.24	0.001	9.97	6.76	14.70	0.001
All extraocular muscles (EOM) enlargement	No	1.00				1.00			
	Yes	3.75	2.58	5.46	0.001	2.89	1.88	4.45	0.001
Age	≤ 30	1.00				1.00			
	31–45	2.47	1.66	3.68	0.001	1.14	0.74	1.75	0.559
	46+	1.71	1.24	2.35	0.001	1.02	0.71	1.46	0.930
Caruncular/plica injection	No	1.00				1.00			
	Yes	0.97	0.75	1.25	0.824	1.03	0.78	1.35	0.858
Chemosis	No	1.00				1.00			
	Yes	0.84	0.64	1.09	0.189	0.97	0.74	1.29	0.849
Lid swelling	No					1.00			
	Yes	0.90	0.70	1.16	0.412	0.94	0.71	1.25	0.692
Pain	No					1.00			
	Yes	1.07	0.82	1.40	0.596	1.05	0.77	1.41	0.776
Retraction	No					1.00			
	Yes	1.04	0.80	1.36	0.753	1.04	0.79	1.37	0.796

**Table 4:** The relationship between all extraocular muscle (EOM) enlargement in the computed tomography (CT) scan and medical intervention time (start time) with the length of the treatment period until the disappearance of all symptoms.

**Parameter**		**Median**	**95% confidence interval (CI)**		***P*-value**
			**Lower**	**Upper**	
Medical intervention time (from the time the diagnosis was made)	≤ 6	6.000	5.375	6.625	<0.001
	7+	12.000	11.345	12.655	
CT scan of all EOM enlargement	No	8.000	6.829	9.171	<0.001
	Yes	18.000	16.671	19.329	

In this study, we evaluated proptosis changes and examined their relationship with other parameters. The results showed that better response (more reduction) in proptosis was associated with: younger age at the start of treatment, earlier treatment intervention (ever since the diagnosis of moderate to severe was made), shorter treatment time (less time to improvement), less IOP, lack of all EOM enlargement on CT scan and lack of diplopia (*P* < 0.05; Table 5).

**Table 5:** Relationship between proptosis changes and the other study parameters.

**Parameter**		**Beta**	**95% confidence interval (CI)**		***P*-value**
			**Lower**	**Upper**	
Sex	M	−0.02	−0.11	0.07	0.701
	F	ref			
Age		0.01	0.00	0.01	0.001
Start time of medication		0.03	0.02	0.04	0.001
Time to improvement		0.02	0.017913	0.032048	0.001
Conjunctival injection		−0.06	−0.14915	0.031844	0.204
Caruncular/plica Injection		0	−0.082	0.090152	0.926
Chemosis		0	−0.09203	0.088293	0.968
Lid swelling		0.02	−0.06885	0.104465	0.687
Pain		0.02	−0.07174	0.109128	0.685
Retraction		0.01	−0.0781	0.101282	0.8
CT scan of all EOM enlargement		−0.42	−0.51395	−0.33087	0.001
Intraocular pressure (IOP) baseline		0.02	0.008447	0.027924	0.001
Diplopia any gaze		−0.21	−0.29606	−0.12159	0.001
Diplopia primary position		−0.13	−0.23021	−0.03528	0.008
Proptosis baseline		0.02	−0.00627	0.040567	0.151

No severe adverse events, exacerbation, or recurrence of symptoms were recorded in the patients treated with MMF and prednisolone. Adverse events occurred in six of 261 patients (excluded from this study). Four patients showed impaired liver function (liver enzymes values slightly more than the upper limit), which recovered after internal medicine consult and hepatoprotective drug treatment. Two patients had gastrointestinal (GI) complications and did not want to continue the study due to old age and were therefore excluded from the study.

## Discussion

To the best of our knowledge, only a few studies have described and evaluated the use of MMF in a real clinical setting for GO treatment. Moreover, this is study to reaffirmation the efficacy and safety of MMF and examine “MMF (CellCept®) + oral prednisolone” as a routine treatment for GO patients and evaluate the factors associated with a better response to this regimen; that is, a reduction in proptosis and time to improvement.

Various studies have evaluated the effect of steroids on GO, and currently, these drugs are the most used in the clinic as a first-line treatment (14, 15). However, due to the high adverse effects of steroids, it is necessary to introduce alternative therapies for these patients. Various studies have shown the effect of immunomodulatory therapies in the treatment of GO, and indicated a new approach in the treatment of this disease, so that immunomodulatory drugs cover most of the treatment (7–9). For example, Kahaly et al. compared the use of mycophenolate combined with methylprednisolone vs. methylprednisolone alone in active, moderate-to-severe Graves' orbitopathy and, showed that the addition of a moderate daily oral dose of mycophenolate (one 360 mg tablet twice per day for 24 weeks) to an established moderate dose of intravenous methylprednisolone (500 mg once per week for 6 weeks, followed by 250 mg per week for 6 weeks) did not significantly affect the rate of response at 12 weeks or rate of relapse at 24 and 36 weeks, but the addition of mycophenolate did lead to significant improvements in the patients' quality of life and ophthalmic symptoms and signs (16). Despite these studies and according to Jiskra, the effects of biologic drugs such as MMF and cyclosporine in TED patients have not been adequately studied, and there is a controversy about their use in the treatment (17).

This prospective interventional case series showed that MMF plus low-dose oral prednisolone as a new treatment regimen was significantly effective in 94.2% of patients, consistent with data previously reported on the effect of MMF on moderate to severe GO (16, 18–21). Similar to the results of teprotumumab (novel insulin-like growth factor I receptor antibody) in active TED (22, 23), as expected, MMF plus low-dose oral prednisolone showed the greatest effectiveness on CAS, proptosis and diplopia, and this factor significantly decreased during the study, and this decrease occurred earlier in CAS than proptosis and diplopia, suggesting that; MMF like teprotumumab was more efficient than corticosteroids in controlling local inflammation. The overall clinical response rate of the patients who received MMF + oral low-dose prednisolone significantly increased with increasing time, confirming that a fixed dosage in a longer duration of oral MMF + oral-low dosage prednisolone might lead to a better response. Our results showed that visual acuity and IOP did not change significantly during the study period. In this regard, Xiaozhen et al. showed that after 24 months of treatment, the response in the group treated with MMF was significantly better than that in the group treated with corticosteroids (91.3 vs. 67.9%, *P* = 0.000), and this study also found that adverse events were more common in the corticosteroid-treated group than in the other groups, and the results showed that the rate of diplopia and proptosis in the group treated with MMF significantly improved compared to that in the group treated with corticosteroids (90.4 and 68.8% improved) (18).

The MMF dosage that was initially used in combination with low-dose prednisolone, according to the study design, was based on a previous work in autoimmunity (24–26). A previous study showed that even low doses of MMF (1,000 mg/d) were effective in suppressing orbital inflammation (16–21). However, in our study, to better respond to MMF, we added the lowest dose of corticosteroids (oral prednisolone, 5 mg/d) to the treatment regimen. With a lower MMF and prednisolone dose, patients would be exposed to lower risks of side effects, such as menstrual disorders, weight gain, and reactivation of infections. In this study, we found significant response rate and fewer adverse events with this treatment regimen. For active GO, a course of low-dose oral MMF (1,000 mg/d) plus low-dose oral prednisolone (5 mg/d) within 12 months can therefore be recommended. However, to prove the effectiveness of this regimen, a clinical trial study and the presence of a control group are required.

Another important finding of our study is, the understanding of the relationship between time to improvement and other study parameters. Our results showed that early onset of drug intervention (under 6 months compared to over 6 months), younger age at disease onset, and lack of all EOM enlargement on CT scan respond faster to treatment; they have less time to improve. Therefore, it seems that the EOM enlargement is one of the reasons for the late response to the treatment regimen. In this regard, Wang et al. showed that patient with fibrotic changes, which cause chronic complications, are difficult to respond to treatment; therefore, early intervention with a proper regimen, especially immune system modulators (primary pathophysiology), can provide a better and faster response (22).

Our study first examined the factors associated with decreased proptosis during GO treatment. Our study showed that a better response (more reduction) in proptosis was related to younger age at disease onset, earlier treatment intervention, shorter treatment duration (less time to improvement), less IOP, lack of EOM enlargement on CT scan, and lack of diplopia. Regarding the better reduction of proptosis with MMF, Ye et al. and Kahaly et al. showed a significant improvement in diplopia and proptosis in the group treated with MMF compared with those in the corticosteroid-treated group (16, 18).

Mycophenolate mofetil is safe and tolerable and the main adverse events of MMF are hematological and GI disorders (26–28). Adverse events associated with steroids include GI effects, glucose intolerance, weight gain, osteoporosis, and skin thinning. Steroids and MMF are potent immunosuppressive and anti-inflammatory drugs; therefore, infections are common and are potentially dangerous adverse effects (28–30). In our study, side effects occurred in six of 261 patients, which was statistically negligible.

The current study had a high number of samples, that is, 242 patients, emphasizing the significance of our findings. In addition, unlike previous studies, we performed a long follow-up observation period of up to 6 months after the therapeutic response (18th month follow-up). However, due to the lack of a control group, the results of our study could not be confirmed nor rejected; thus, it is necessary to conduct trial studies in the future.

In conclusion, MMF combined with low-dose prednisolone may be considered for GO treatment due to its better therapeutic effect and fewer adverse reactions compared with those of other treatment strategies. This regimen may be introduced as a novel, comprehensive, safer, and more effective regimen for chronic complications such as proptosis and diplopia. Further high-quality clinical trials with large sample sizes, are needed to confirm these results and to evaluate the efficacy and safety of MMF (CellCept®) plus low-dose prednisolone for the treatment of active GO.

## Data Availability Statement

The raw data supporting the conclusions of this article will be made available by the authors, without undue reservation.

## Ethics Statement

The studies involving human participants were reviewed and approved by Ethical Committee of Tehran University of Medical Sciences, Ethics Code: IR.TUMS.FARABIH.REC.1397.053. The patients/participants provided their written informed consent to participate in this study.

## Author Contributions

MTR, SR, and BE contributed to conception and design of the study. NM, SH, and MBR organized the database. MK and MS performed the statistical analysis. AM wrote the first draft of the manuscript. MTR, SP, and MP wrote sections of the manuscript. All authors contributed to manuscript revision, read, and approved the submitted version.

## Conflict of Interest

The authors declare that the research was conducted in the absence of any commercial or financial relationships that could be construed as a potential conflict of interest.

## Publisher's Note

All claims expressed in this article are solely those of the authors and do not necessarily represent those of their affiliated organizations, or those of the publisher, the editors and the reviewers. Any product that may be evaluated in this article, or claim that may be made by its manufacturer, is not guaranteed or endorsed by the publisher.
